# Long-term high-risk drinking does not change effective doses of propofol for successful insertion of gastroscope in Chinese male patients

**DOI:** 10.1186/s12871-022-01725-2

**Published:** 2022-06-16

**Authors:** Pei-Pei Hao, Tian Tian, Bin Hu, Wei-Chao Liu, Ying-Gui Chen, Tian-Yu Jiang, Fu-Shan Xue

**Affiliations:** Department of Anesthesiology, Beijing Friendship Hospital, Capital Medical University, NO. 95 Yong-An Road, Xi-Cheng District, Beijing, 100050 People’s Republic of China

**Keywords:** Long-term high-risk drinking, Propofol, Effective dose, Gastroscopy, Sedation

## Abstract

**Background:**

Available literature indicates that long-term drinkers demand a higher dose of propofol for induction of anesthesia than non-drinkers. However, there is no study having assessed the influence of long-term high-risk drinking (LTHRD) on the effective doses of propofol for successful insertion of gastroscope with sedation. This study was designed to compare the effective doses of propofol for successful insertion of gastroscope between LTHRD and non-drinking (ND) Chinese male patients.

**Methods:**

Thirty-one LTHRD patients and 29 ND male patients undergoing elective gastroscopy with propofol sedation were enrolled. The modified Dixon’s up-and-down method was applied to determine the calculated median effective dose (ED_50_) of propofol for successful insertion of gastroscope. Furthermore, the isotonic regression analysis was used to establish the dose–response curve of propofol and assess the effective doses of propofol where 50% (ED_50_) and 95% (ED_95_) of gastroscope insertions were successful.

**Results:**

The calculated ED_50_ of propofol for successful insertion of gastroscope was 1.55 ± 0.10 mg/kg and 1.44 ± 0.11 mg/kg in the LTHRD and ND patients. The isotonic regression analysis further showed that ED_50_ and ED_95_ of propofol for successful insertion of gastroscope was 1.50 mg/kg (95%CI, 1.40–1.63) and 1.80 mg/kg (95%CI, 1.74–1.90) in the LTHRD patients, respectively; 1.40 mg/kg (95% CI, 1.27–1.57) and 1.60 mg/kg (95%CI, 1.56–1.65) in the ND patients. The ED_50_ of propofol for successful insertion of gastroscope was not significantly different between LTHRD and ND patients.

**Conclusions:**

This study demonstrates that the difference in the estimated ED_50_ of propofol for successful insertion of gastroscope between LTHRD and ND Chinese male patients was not statistically significant.

**Trial registration:**

The study was registered on November 28, 2020 (ChiCTR2000040382) in the Chinese Clinical Trial Registry.

**Supplementary Information:**

The online version contains supplementary material available at 10.1186/s12871-022-01725-2.

## Background

The latest reports released by World Health Organization (WHO) show that the number of drinkers has reached 2.3 billion globally and alcohol addiction has become a leading risk factor for personal death and disability [1]. In China, the number of regular heavy drinkers also has been increasing rapidly [2]. The risk levels of drinking are commonly defined by the WHO criteria, in which a low-risk drinking level for men and women is defined as alcohol consumption between 1–40 g/day and between 1–20 g/day, respectively; a medium-risk drinking level as between 41–60 g/day and between 21–40 g/day, and a high-risk drinking level as more than 61 g/day and more than 41 g/day [3].

Individuals with alcohol consumption are commonly comorbid with liver disease, such as alcoholic hepatitis and liver cirrhosis [4]. As the liver is a main organ that is involved in drug metabolism, hepatic insufficiency can significantly change the metabolism of anesthetic and analgesic drugs [5, 6]. Furthermore, available evidence indicates that alcohol consumption is closely related to the development of digestive tract diseases, such as esophageal cancer [1, 3], gastritis [3], esophagitis and/or Barrett’s esophagus [7], duodenal ulcers [8], and others. Thus, the requirement of painless gastroscopy by long-term drinkers has been rising for early diagnosis and treatment of upper gastrointestinal diseases [9].

As propofol is a sedative agent with unique pharmacological properties including rapid onset and satisfactory postoperative recovery, it is commonly used for sedation of painless gastroscopy [10]. It has been shown that long-term drinkers compared with no drinkers demand an increased dose of propofol for anesthetic induction [11, 12]. Furthermore, the pharmacokinetics of propofol are mildly different between long-term drinking and non-drinking patients [13]. This suggests that long-term drinking may change the effective doses of propofol for sedation of painless gastroscopy. However, there is no study having assessed the influence of long-term drinking on the effective doses of propofol for sedation of painless gastroscopy. Thus, this study was designed to compare the effective doses of propofol for successful insertion of gastroscope between long-term high-risk drinking (LTHRD) and non-drinking (ND) patients by the modified Dixon’s up-and-down method (MDUDM).

## Methods

The protocol of this clinical study was approved by the Ethics Committee of Beijing Friendship Hospital, Capital Medical University (2020-P2-237–01) and was registered in the Chinese Clinical Trial Registry (ChicTR2000040382). This study enrolled the LTHRD and ND male outpatients undergoing elective single gastroscopy without therapeutic procedure under propofol sedation, with age of 35 to 65 years, American Society of Anesthesiologists (ASA) physical status classification I-II and body mass index (BMI) of less than 30 kg/m^2^. The exclusion criteria were hepatic and renal dysfunction, liver cirrhosis, allergy to propofol, a history of long use of sedative or antipsychotic drugs, alcohol abstinence, signs of alcohol addiction (such as inability to reduce alcoholic consumption despite obvious deleterious effects and morning tremor alleviated by an alcoholic beverage) [13], and not willing or able to finish the study. The written informed consent was obtained from each patient who agreed to participate in the study. Furthermore, this study was conducted in accordance with the Basic and Clinical Pharmacology and Toxicology policy for experimental and clinical studies [14].

Before gastroscopy, patients were screened by a face-to-face questionnaire. The criteria for definition of LTHRD were pure alcohol consumption greater than 61 g/day (high-risk level and above) for at least 2 years or more [3, 11, 12]. The criteria for definition of ND were never or occasional drinking. The alcohol content of drinks depends on the strength of the beverage and volume of container. Furthermore, the volume of alcohol in any drink can be converted into grammes of ethanol by following formula: contents of ethanol (g) = volume (ml) × strength of beverage (vol%) × conversion factor (0.8) [15]. According to Chinese market situation, a conversion table was made to easily calculate the content of ethanol in drinks that patients consumed during the study (Supplemental Table 1). Once patients were defined as LTHRD, the liver function tests including ALT and AST were examined to exclude significant liver dysfunction.

**Table 1:** The demographic and medical data of patients

	LTHRD group (*n* = 31)	ND group (*n* = 29)	*P* values
Age (yrs.)	53.6 ± 7.0	51.0 ± 11.4	0.290
Height (cm)	172.0 ± 5.1	172.7 ± 5.2	0.610
Weight (kg)	76.7 ± 10.5	73.7 ± 9.4	0.238
BMI (kg/m^2^)	25.8 ± 2.7	24.7 ± 2.1	0.086
Mallampati grades			
I	13(41.9%)	13(44.8%)	0.688
II	12(38.7%)	12(41.4%)	
III	6(19.4%)	4(13.8%)	
ASA classifications			
I	9(29.0%)	17(58.6%)	0.022
II	22(70.1%)	12(41.4%)	
ALT(U/L)	20.3 ± 7.8	25.1 ± 9.5	0.035
AST(U/L)	24.7 ± 10.7	24.0 ± 14.0	0.806
GGT(mmol/L)	52.1 ± 27.0	27.6 ± 15.4	*P* ≤ 0.001
Cr (µmol/L)	76.6 ± 17.6	81.1 ± 17.8	0.327
Alcohol consumption (g/d)	75.0 ± 14.3	-	-
Duration of alcohol (yrs)	24.7 ± 11.2	-	-
Cigarettes			
Yes	17(54.8%)	5(17.2%)	0.003
No	14(45.2%)	24(82.8%)	

*LTHRD* Long-term high-risk drinking, *ND* No-drinking, *ASA* American Society of Anesthesiologists, *ALT* Alanine aminotransaminase, *AST* Aspertate aminotransferase, *Cr* Creatinine, *GGT* γ-glutamyl transpeptidase, *BMI* Body mass index

All participants were fasted from solids for 8 h and liquids for 2 h and did not receive any premedication. After patients entered the endoscopy room, an intravenous access was obtained in the arm and the lactate ringer’s solution was infused. The routine monitoring, including noninvasive blood pressure, electrocardiogram, and pulse oxygen saturation (SpO_2_) (Kuotaneenkatu 2 FI-00510 Helsinki, Finland), was applied. According to our routine practice, patients were positioned in a left lateral position and the head was supported by a pillow to align the axis of the head, neck, and trunk in line. Preoxygenation was performed with 6 L/min oxygen through a facemask which was held by patient. Patient was instructed to take deep breaths for at least 2 min before propofol administration. During the endoscopy, oxygen facemask was placed near patient’s nose.

According to our previous experience on propofol mono-sedation for gastroscopy [16], an initial dose of propofol (Corden Pharma S.P.A. Viale dell’ Industria 3, 20,867 Caponago, Italy. RL708) for the first patient was set at 1.6 mg/kg according to total body weight, which was prepared and administered intravenously in 30 s to induce sedation by anesthetist A. Then, anesthetist B evaluated the sedation depth every 2 min by the Ramsay sedation scale (Supplemental Table 2) [17]. The anesthetist B was well trained to master application of the Ramsay sedation scale before the initiation of this study. The syringe was wrapped by black paper to ensure that both anesthetist B and endoscopist were blinded to the propofol dosage. When peak effect of propofol was reached at 2 min after intravenous injection or anesthetist B determined that a Ramsay’s score of 5 was achieved, gastroscope insertion was attempted by an experienced attending physician, who had performed at least 3000 gastroscopy examinations before this study. The responses of patient to gastroscope insertion were evaluated by anesthetist B and categorized as either “movement” or “no movement”. Both anesthetist B and endoscopist were also blinded to the group assignment of patients. According to the MDUDM [18], the initial dose of propofol for the next patient was determined by previous patient’s responses, with an increment of propofol 0.1 mg/kg (if “movement” responses occurred in the previous patient) or a decrement of propofol 0.1 mg/kg (if “no-movement” responses occurred in the previous patient). Anesthetist A calculated and prepared the propofol dose of the next patient according to the responses of patient provided by anesthetist B. A crossover point was defined when patient’s responses were transformed from “movement” to “no movement” or from “no movement” to “movement”. To obtain the stable estimates for effective dose of a drug, the MDUDM commonly requires that at least 6 crossover points are obtained and 20 to 40 subjects are included [19–21]. After 7 crossover points had been obtained and about 30 patients in each group were included, thus, our patient recruitment was stopped.

**Table 2:** Observed and pool adjacent violators algorithm (PAVA) response rates, indicating the proportion of successful gastroscope insertion in the long-term high-risk drinkers with each dose of propofol

Dose	Number of successes	Number of patients	Observed responses	PAVA-adjusted response rates
1.3	0	3	0.000	0.000
1.4	3	8	0.375	0.375
1.5	5	10	0.500	0.500
1.6	6	7	0.857	0.778
1.7	1	2	0.500	0.778
1.8	1	1	1.000	1.000

After the initial dose of propofol was administered, the endoscopy would not begin until a Ramsay score greater than 4. The “movement” responses were defined as retching, cough, and/or limb movement to refuse gastroscope insertion. If the Ramsay’s score was less than 5 at 2 min after the initial dose of propofol, additional dose of propofol 20–30 mg was administered over 30 s and this situation was categorized as the “movement” responses. If the “movement” responses to gastroscope insertion were determined, a bolus dose of propofol 20–30 mg was also administered intravenously. After completion of endoscopic procedure, all patients were transferred into the post-anesthesia recovery room for close observation until the criteria of hospital discharge were reached.

Blood pressure (BP), heart rate (HR), and SpO_2_ were recorded before drug administration (baseline, T1), at the beginning of endoscopy (T2), 2 min after the beginning of endoscopy (T3), and the end of endoscopy (T4). All circulatory and respiratory adverse events occurring during the observed period, including respiratory depression, hypotension, bradycardia, were recorded. Respiratory depression was categorized into subclinical oxygen desaturation (SpO_2_ = 90 to 95%), oxygen desaturation (SpO_2_ = 75 to 89% for < 60 s), severe oxygen desaturation (SpO_2_ < 75% at any time, or 75 ≤ SpO_2_ < 90% for > 60 s) [22]. If oxygen desaturation (SpO_2_ < 90%) occurred, simple airway manipulation, such as chin lift or jaw thrust, was first used. If SpO_2_ was not improved, facemask ventilation with 100% oxygen was provided. Hypotension, which was defined as systolic blood pressure (SBP) less than 90 mmHg, was treated with intravenous ephedrine 6 mg. Bradycardia, which was defined as HR less than 50 beats/min), was treated with intravenous atropine 0.5 mg [23].

After endoscopic examination, the endoscopist was asked to assess both the ease of gastroscope insertion via the upper airway (rank: easy/medium/hard) and the satisfaction with entire sedation (rank: satisfied/medium/dissatisfied). At 24 h after endoscopic examination, a telephone follow-up was performed to determine patient’s satisfaction with endoscopy under sedation, evaluate the occurrence of nausea and vomiting and enquire patients about their willingness to undergo the next gastroscopy with same sedation management. In order to ensure that patients accurately evaluated their satisfaction with endoscopy under sedation, they were instructed how to use the designed rank criteria (satisfied/medium/dissatisfied) before examination.

### Statistical analysis of data

Data were analyzed using the Statistical Package for Social Sciences (SPSS, software Version 26.0). Continuous variables were presented as mean ± SD and categorical variables were presented as number and/or percentage. The Kolmogorov–Smirnov test was applied to exam data distribution. For intergroup comparison of normal distributed data, an independent Student’s t-test was used, and for intergroup comparison of data with non-normal distribution, a Mann–Whitney U test was used. For categorical variables, cross-tabulation, and Pearson’s χ^2^ test were applied. A *P* value less than 0.05 was considered statistically significant.

The median effective dose (ED_50_) of propofol was calculated by the mean of midpoints of all crossover points from “movement” responses to “no movement” responses according to the MDUDM [19]. Because of patient variability, there is not always consistent with the assumption in the MDUDM that the drug effect is monotonic increasing with an increasing dose. Thus, a pooled-adjacent-violators algorithm (PAVA) was used to ensure a monotonic response rate [19, 24]. With the “R” package (R version 4.1.1 2021–08-10), moreover, the isotonic regression analysis was performed to establish the dose–response curves of propofol and obtain the effective doses of propofol where 50% (ED_50_) and 95% (ED_95_) of gastroscope insertions were successful, and their 95% confidence intervals (CI) [25]. According to the method reported in previous work [24], the overlapping CI method was used to compare the difference in ED_50_ estimated by the isotonic regression analysis between LTHRD and ND groups. After 83% CI of ED_50_ is obtained, if the 83% CI is overlap, then the null hypothesis of equal ED_50_ is rejected at a significance level α of approximately 0.05.

## Results

From December 2020 to January 2021, a total of 60 patients were enrolled into the study. Of 60 participants, 31 were LTHRD and 29 were ND. The demographic and medical data of patients were shown in Table 1 and were not significantly different between LTHRD and ND patients, except for γ-glutamyl transpeptidase, alanine aminotransaminase, ASA physical status classifications, and smoking history.

**Fig. 1:**
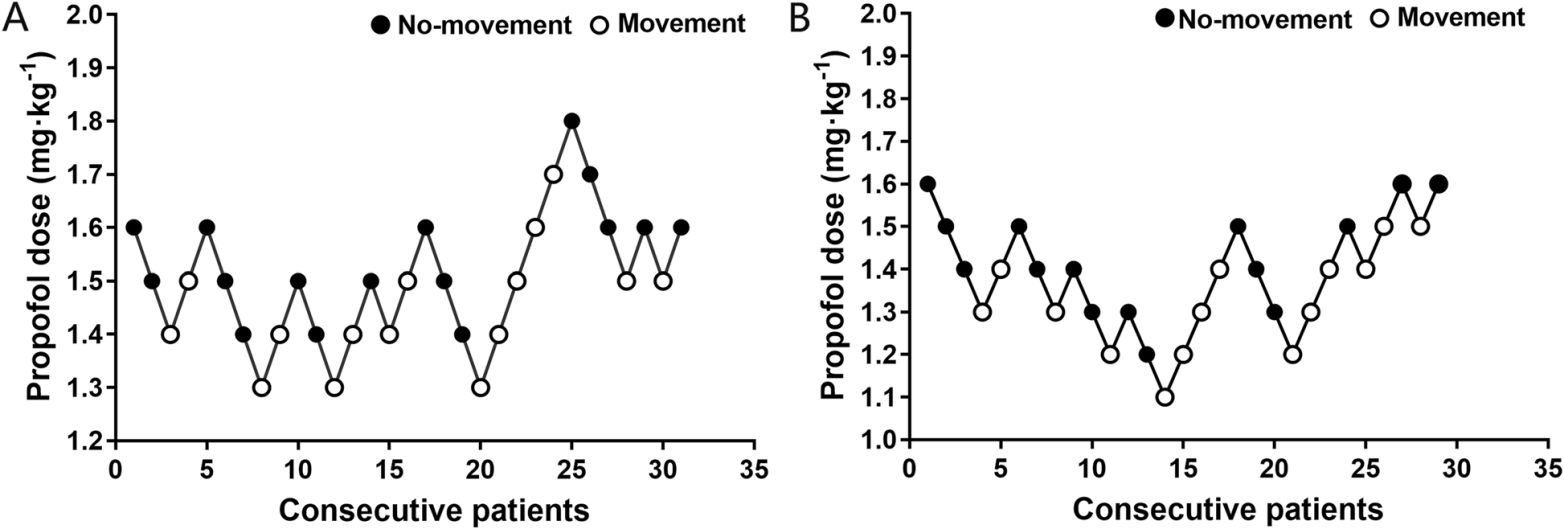
Responses of LTHRD (**A**) and ND (**B**) patients to propofol with the modified up-and-down method. LTHRD, Long-term high-risk drinking; ND, no-drinking

The group allocations and responses of patients to propofol with the MDUDM are represented in Fig. 1, and Tables 2 and 3. Based on the mean of midpoints of all crossover points from “movement” responses to “no movement” responses, the calculated ED_50_ of propofol for successful insertion of gastroscope were l.55 ± 0.10 mg/kg and 1.44 ± 0.11 mg/kg in the LTHRD and ND patients, respectively, without a statistically significant difference between groups (*P* = 0.06).

**Table 3:** Observed and pool adjacent violators algorithm (PAVA) response rates, indicating the proportion of successful gastroscope insertion in the non-drinkers with each dose of propofol

Dose	Number of successes	Number of patients	Observed responses	PAVA-adjusted response rates
1.1	0	1	0.000	0.000
1.2	1	4	0.250	0.250
1.3	3	7	0.428	0.428
1.4	4	8	0.500	0.500
1.5	4	6	0.667	0.667
1.6	3	3	1.000	1.000

**Fig. 2:**
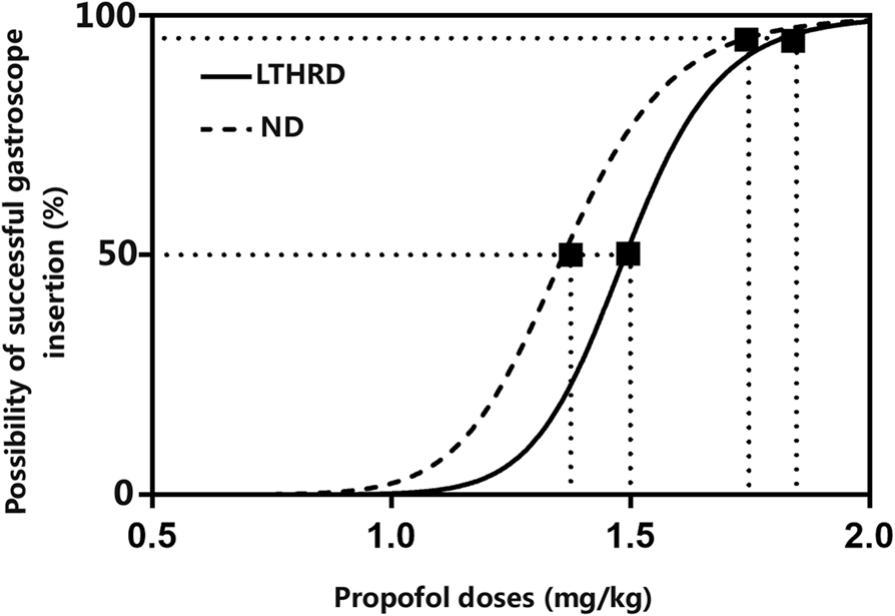
Dose–response curves of propofol for successful insertion of gastroscope. LTHRD, Long-term high-risk drinking; ND, no-drinking

The dose–response curves of propofol for successful insertion of gastroscope plotted from the isotonic regression are shown in Fig. 2. The dose–response curve of propofol was slightly shifted to the right in the LTHRD patients compared to the ND patients. According to the isotonic regression analysis, ED_50_ and ED_95_ of propofol for successful insertion of gastroscope was 1.5 mg/kg (95%CI, 1.40–1.63; 83% CI 1.44–1.60) and 1.8 mg/kg (95%CI, 1.75–2.05) in the LTHRD patients, respectively; 1.4 mg/kg (95% CI, 1.27–1.57; 83% CI 1.31–1.53) and 1.6 mg/kg (95% CI, 1.54–1.69) in the ND patients. The ED_50_ and ED_95_ of propofol for successful insertion of gastroscope were somewhat increased in the LTHRD patients. Based on the 83% CI of ED_50_, however, the overlapping CI method showed that the estimated ED_50_ by the isotonic regression analysis was not statistically different between groups (*P* > 0.05). Furthermore, total average doses of propofol required for completion of gastroscopy in per patient were 178.7 ± 35.8 mg and 160.5 ± 39.4 mg in the LTHRD and ND groups, respectively, without a statistically significant difference between groups (*P* = 0.066).

The changes of HR, SpO_2_, and MAP, and the occurrence of adverse events during the observation are shown in Supplemental Table 3. As compared to baseline at T1, MAPs at T2, T3, and T4 decreased significantly in the two groups (*P* < 0.05). The MAP in T4 was significantly lower in the ND patients than in the LTHRD patients (*P* < 0.05). The incidence of subclinical oxygen desaturation was 32.3% and 34.5% in the LTHRD and ND patients, respectively, without a statistically significant difference (*P* > 0.05). One case in each group presented oxygen desaturation. The incidence of hypotension was 9.7% and 7.9% in the LTHRD and ND patients, respectively. The other adverse events such as nausea and vomiting were not observed.

The gastroscopy time was 389.9 ± 168.7 s and 395.2 ± 209.3 s in the LTHRD and ND patients, respectively, without a statistically significant difference between groups. Furthermore, satisfactions of patients and endoscopists and ease of gastroscope insertion were not significantly different between groups (Supplemental Table 4).

## Discussion

The number of drinkers worldwide is growing rapidly [1], but the influence of long-term drinking on the effective doses of propofol for sedation of painless gastroscopy remains unclear. Thus, this study assessed the effective doses of propofol for successful insertion of gastroscope in the LTHRD and ND Chinese male patients and determined if the effective doses of propofol for successful insertion of gastroscope were significantly different between them.

During this study, the MDUDM was firstly used to determine the calculated ED_50_ of propofol for successful insertion of gastroscope. In clinical pharmacological study, moreover, the logistic or probit regression analysis is also commonly used approach to estimate the effective doses of a drug. As the assigned dose values of a drug in the MDUDM are nonindependence, however, the logistic or probit regression analysis may lead to a biased regression slope, i.e., a much steeper dose–response curve than expected is often obtained [19, 24, 25]. The isotonic regression analysis is a well-described variant of restricted least-squares regression that contains an implicit assumption, which is drug effect increasing with an increasing dose [19]. Recently, it is recommended that effective doses of a drug should be estimated by using the isotonic regression analysis, in which the bias is small and a more accurate targeted dose can be provided [26]. Thus, in this study, the isotonic regression analysis was used to further estimate the ED_50_ and ED_95_ of propofol for successful insertion of gastroscope. Our results showed that the effective doses of propofol for successful insertion of gastroscope was slightly increased and the dose-response curve was somewhat shifted to right in LTHRD patients compared to ND patients. However, the overlapping CI method showed that the between-group difference of ED_50_ of propofol for successful insertion of gastroscope did not reach the level of statistical significance. In fact, according to the results of isotonic regression analysis, the net differences in ED_50_ and ED_95_ of propofol for successful insertion of gastroscope between LTHRD and ND patients are only 0.1–0.2 mg/kg. Furthermore, the total doses of propofol required for the completion of gastroscopy were not significantly different between LTHRD and ND patients. All of these indicate that long-term drinking does not significantly change the effective dose of propofol for sedation of painless gastroscopy. Our results are in accord with the findings of Servin et al.’s study [13], in which long-term drinking does not significantly affect the requirement of propofol for laryngoscopy under general anesthesia.

However, other studies reported the different findings. Liang et al. [11] demonstrated that when anesthesia was induced by target-controlled infusion of propofol and remifentanil, the effective concentrations 50% (EC_50)_ and 95% (EC_95_) of propofol at loss of consciousness in the alcoholic patients were 3.15 (95% CI, 2.77–3.37) and 4.05 (95% CI, 3.18–5.26) μg/ml, respectively, which were significantly higher than those in the control patients [2.21 (95%CI, 1.92–2.86) and 3.04 (95%CI, 2.45–4.64) μg/ml]. In the study of Fassoulaki et al. [12], moreover, the doses of propofol required to produce loss of verbal contact and loss of ability to grasp a 20-ml syringe filled with water were significantly increased in the alcoholic patients (2.7 ± 0.42 and 4.2 ± 1.02 mg/kg) compared with the control patients (2.2 ± 0.43 and 3.2 ± 0.75 mg/kg). These findings indicate that the effective doses of propofol required for anesthesia induction are significantly increased in chronic alcoholic patients.

The exact causes for these contradictory results of our and other studies are unclear, but the differences among studies in the study objects, sex and/or age, methods of propofol administration, tested procedures and targeted depth of sedation or anesthesia may be attributable to these various findings. First, our study objects are the LTHRD patients, who are defined as alcohol consumption of at least 61 g/d. Furthermore, patients with alcohol addiction are excluded from our study, as their more complex conditions may lead to biased research results. In addition, alcohol withdrawal symptoms, such as tremulousness, sweating, gastric distress and weakness, often start within 8 h of abstention, while painless endoscopic procedure is also often performed after 6–8 h of fasting. Especially, severe alcohol withdrawal symptoms including hypertension, hyperreflexia and hallucinations may even occur after a delay of up to five days [27]. Evidently, these issues may also increase difficulties on clinical management of painless endoscopic procedure. In contrast, the objects of Liang et al.’ study [11] are patients with chronic alcoholism, who are defined as alcohol consumption greater than 75 g/d with the symptoms of alcohol addiction and biologic abnormalities of alcohol abuse such as raised γ-glutamyl transpeptidase level and increased mean corpuscular volume of erythrocytes. Similarly, Fassoulaki et al.’s study [12] was also performed in patients with chronic alcoholism, who are defined as alcohol consumption greater than 40 g/d with abnormal γ-glutamyl transpeptidase level and increased mean corpuscular volume of erythrocytes. Second, the targeted sedation depth in our study is a Ramsay’s score of about 5. In the studies of Liang et al. [11] and Fassoulaki et al. [12], a deeper sedation, i.e., loss of consciousness, is required. A deeper depth of sedation means the need of a larger propofol dose. With a larger dose of drug, it perhaps is easier to exhibit differences in the effective doses of propofol between patients with and without alcohol consumption. Third, Fassoulaki et al.’s study included 4 women and 25 men [12], while our study only enrolled male patients., clinical studies demonstrate that women require more propofol to maintain a similar level of anesthesia and recover faster from anesthesia than men [28–30]. Fourth, the studies of Liang et al. [11] and Fassoulaki et al. [12] include the patients with definited hepatic dysfunction. In our study, serum levels of γ-glutamyl transpeptidase and alanine aminotransaminase were significantly higher in the LTHRD patients than in the ND patients, but they did not meet the criteria of hepatic dysfunction. Fifth, the mean ages of alcoholic and control patients are 46.2 and 47.5 years in Liang et al.’ study [11], respectively, and 48 and 38 years in Fassoulaki et al.’ study [12]. That is, the subjects of our study are older than those of Liang et al.’ and Fassoulaki et al.’ studies. It has been shown that age can significantly affect the clinical pharmacodynamics of propofol, i.e., increasing age results in a decreased requirement of propofol [31, 32].

Because of a rapid onset and a short recovery time, propofol is one of sedative agents commonly used for painless endoscopy requiring sedation or anesthesia. Available evidence indicates that both endoscopists and patients are very satisfied with propofol sedation for endoscopy [33, 34]. In our study, patients in the two groups were highly satisfied with propofol sedation, but the endoscopists’ satisfaction rated as “excellent” were only 67.7% and 65.5% in the LTHRD and ND patients, respectively. This poor satisfaction of endoscopists may be attributable to propofol administration according to the requirements of the MDUDM, which are used to determine the minimal effective dose of propofol.

There have been a lot of clinical studies about the effective doses of propofol for endoscopic procedures. For example, in healthy adult patients aged 18–65 years, Liu et al. [16] demonstrate that ED_50_ of propofol mono-sedation for successful insertion of gastroscope is 1.89 mg/kg, which is slightly higher than that of ND patients in our study. These diverse findings may be attributable to the differences in intravenous administration rates of initial propofol, which are 20 s and 30 s in Liu et al.’s and our studies, respectively. It has been shown that a slow rate of intravenous administration can significantly reduce the requirement of propofol [35]. Furthermore, the subjects of study are younger in Liu et al.’s study compared with our study.

Our study design has some potential limitations that deserve special attention. First, the depth of sedation was not monitored by automatic electroencephalogram analysis and only the Ramsay scale was used to assess the depth of sedation. This may produce a subjective bias in the assessment of sedation depth. Second, all participants in our study were patients aged 35-65 years, because there are seldom long-term drinkers who are age less than 35 years and present for gastroscopy in our center. Given that age is an important factor affecting pharmacodynamics of propofol [28, 36], our results may be not suitable for the LTHRD patients aged less than 18 years or larger than 65 years. Third, as female long-term drinkers are rare in China and gender is a factor affecting pharmacodynamics of propofol [28–30], our study only included LTHRD male patients. Thus, an important issue that our study cannot answer is whether LTHRD of female patients can significantly change the effective doses of propofol for successful insertion of gastroscope. Fourth, this study only included the LTHRD patients without clinical manifestations of alcohol addiction and hepatorenal dysfunction. Evidently, our results cannot be extrapolated to the patients with clinical manifestations of alcohol addiction and hepatorenal dysfunction. Thus, further studies to address above issues are still needed. Finally, this study is a clinical pharmacological trial comparing the effective doses of propofol between two conditions and is not a randomized controlled trial comparing efficacy of propofol. Thus, the results of this study should be further confirmed by the large-scale, multi-center, randomized, controlled trials.

## Conclusions

This study demonstrates that the differences in the estimated ED_50_ of propofol for successful insertion of gastroscope between LTHRD and ND Chinese male patients were not statistically significant.

## Supplementary Information

**Additional file 1: Supplemental Table 1.** The calculation of ethanol in drinks. **Supplemental Table 2.** Ramsay sedation scale^16^. **Supplemental Table 3.** The changes of HR, SpO_2_ and MAP, and the occurrence of adverse events during observation. **Supplemental Table 4.** The satisfaction of patients and endoscopists.

## Data Availability

The datasets used and/or analyzed during the current study are available from the corresponding author on reasonable request.

## Abbreviations

LTHRD: Long-term High-Risk Drinking
ND: Non-Drinking
ED: Effective Dose
WHO: World Health Organization
ASA: American Society of Anesthesiologists
BMI: Body Mass Index
SpO_2_: Pulse Oxygen Saturation
BP: Blood pressure
HR: Heart Rate
SBP: Systolic Blood Pressure
CI: Confidence Intervals
PAVA: Pooled-Adjacent-Violators Algorithm
MDUDM: Modified Dixon’s Up-and-Down Method
SPSS: Statistical Package for Social Sciences
EC: Effective Concentration
ALT: Alanine Aminotransaminase
AST: Aspertate Aminotransferase
Cr: Creatinine
GGT: γ-Glutamyl transpeptidase
