# Supplementary material for: Long-term high-risk drinking does not change effective doses of propofol for successful insertion of gastroscope in Chinese male patients
Source: BMC Anesthesiol. 2022 Jun 16;22:183. doi: 10.1186/s12871-022-01725-2 (PMC9202194; doi:10.1186/s12871-022-01725-2)
Supplement: Supplementary file 1 — Additional file 1: Supplemental Table 1. The calculation of ethanol in drinks. Supplemental Table 2. Ramsay sedation scale16. Supplemental Table 3. The changes of HR, SpO2 and MAP, and the occurrence of adverse events during observation. Supplemental Table 4. The satisfaction of patients and endoscopists. [file 12871_2022_1725_MOESM1_ESM.docx]

**Legends**

**Supplemental table 1** The calculation of ethanol in drinks

**Supplemental table 2** Ramsay sedation scale^20^

**Supplemental table 3** The changes of HR, SpO_2_ and MAP, and the occurrence of adverse events during observation

**Supplemental table 4** The satisfaction of patients and endoscopists

**Supplemental table 1** The calculation of ethanol in drinks

| **Volume(ml)** | **Beverage(vol%）** | | | | | | |
| --- | --- | --- | --- | --- | --- | --- | --- |
|  | 5 | 28 | 33 | 38 | 43 | 48 | 53 |
|  | **Contents of ethanol (g)** | | | | | | |
| 50 | 2.0 | 11.2 | 13.2 | 15.2 | 17.2 | 19.2 | 21.2 |
| 100 | 4.0 | 22.4 | 26.4 | 30.4 | 34.4 | 38.4 | 42.4 |
| 150 | 6.0 | 33.6 | 39.6 | 45.6 | 51.6 | 57.6 | 63.6 |

**Supplemental table 2** Ramsay sedation scale^17^

| **Score** | **Level of sedation** |
| --- | --- |
| 1 | Patient is anxious and agitated or restless, or both |
| 2 | Patient is co-operative, oriented, and tranquil |
| 3 | Patient responds to commands only |
| 4 | Patient exhibits brisk response to light glabellar tap or loud auditory stimulus |
| 5 | Patient exhibits a sluggish response to light glabellar tap or loud auditory stimulus |
| 6 | Patient exhibits no response |

**Supplemental table 3** The changes of HR, SpO_2_ and MAP, and the occurrence of adverse events during observation

|  | **LTHRD group** | **ND group** | **P values** |
| --- | --- | --- | --- |
| HR (beats/min) |  |  |  |
| T1 | 75.7±12.2 | 81.0±12.5 | 0.098 |
| T2 | 79.4±12.2 | 82.8±10.7 | 0.252 |
| T3 | 78.3±11.7 | 76.2±18.6 | 0.609 |
| T4 | 77.5±13.0 | 81.1±10.6 | 0.253 |
| SpO_2_ (%) |  |  |  |
| T1 | 96.8±1.8 | 97.0±2.4 | 0.751 |
| T2 | 97.5±2.1 | 97.7±2.4 | 0.727 |
| T3 | 96.8±1.9 | 96.7±1.9 | 0.918 |
| T4 | 96.2±2.1 | 96.3±2.4 | 0.838 |
| MAP (mmHg) |  |  |  |
| T1 | 98.5±9.1 | 98.9±9.3 | 0.874 |
| T2 | 85.9±8.8* | 87.2±8.9* | 0.570 |
| T3 | 85.8±10.4* | 83.4±11.7* | 0.393 |
| T4 | 84.6±9.7* | 79.2±10.7* | 0.044 |
| Adverse events |  |  |  |
| Subclinical oxygen desaturation | 10(32.3%) | 10(34.5%) | >0.05 |
| Oxygen desaturation | 1(3.2%) | 1(3.4%) | >0.05 |
| Hypotension | 3(9.7%) | 2(7.9%) | >0.05 |

The data was displayed by mean ± SD or rates. *Compared to T1 time point, P<0.05.

LTHRD, long-term high-risk drinking; ND, no-drinking; T1, before drug administration; T2, the beginning of endoscopy; T3, 2 min after the beginning of endoscopy; T4, the end of endoscopy; HR heart rate; SpO_2_, pulse oxygen saturation, MAP, mean arterial pressure.

**Supplemental table 4** The satisfactions of patients and endoscopists

|  | **LTHRD group (n=31)** | | **ND group (n=29)** | ***P* values** |
| --- | --- | --- | --- | --- |
| Patients’ satisfaction  Satisfied | 31(100%) | 29(100%) | | >0.05 |
| Endoscopist’ satisfaction |  |  | | >0.05 |
| Excellent | 21(67.7%) | 19(65.5%) | |  |
| Medium | 10(32.3%) | 8(27.6%) | |  |
| Bad | 0 | 2(6.9%) | |  |
| The ease of gastroscope insertion | | | | |
| Easy | 23(74.2%) | 23(79.3%) | | 0.05 |
| Medium | 8(25.8) | 5(17.2%) | |  |
| Hard | 0 | 1(3.5%) | |  |

LTHRD, long-term high-risk drinking; ND, no-drinking.
